# Beyond Single-Pollutant Toxicity: How Erythromycin–Metolachlor Mixtures Affect the Physiology of *Raphidocelis subcapitata*

**DOI:** 10.3390/toxics14080645

**Published:** 2026-07-23

**Authors:** Beatriz C. Rocha, Manuela D. Machado, Eduardo V. Soares

**Affiliations:** 1 CIETI-Bioengineering Laboratory, ISEP, Polytechnic of Porto, Rua Dr. António Bernardino de Almeida, 4249-015 Porto, Portugal; bea.rocha2000@hotmail.com; 2 CEB—Centre of Biological Engineering, University of Minho, Campus de Gualtar, 4710-057 Braga, Portugal; 3 LABBELS—Associate Laboratory, 4710-057 Braga, Portugal; 4 LABBELS—Associate Laboratory, 4800-058 Guimarães, Portugal

**Keywords:** erythromycin–metolachlor, algae physiology, growth inhibition, photosynthetic activity, pigments, mixture interactions

## Abstract

This work aimed to evaluate the combined effects of an antibiotic (erythromycin, ERY) and an herbicide (metolachlor, MET), at environmentally relevant concentrations, on the physiology of the freshwater microalga *Raphidocelis subcapitata*. For this purpose, firstly, the alga was exposed to each toxic compound to determine the respective effect concentrations after 72 h (72h-EC values). Subsequently, the alga was exposed to mixtures composed of 72h-EC_10_, 72h-EC_25_, and 72h-EC_50_ values of each toxicant, and interaction analysis was conducted using the independent action (IA) model. Overall, the mixtures of ERY and MET inhibited algal growth synergistically. Regarding photosynthetic performance, exposure to the mixtures of toxicants, generally resulted in a reduction of the maximum photochemical efficiency of photosystem II (PSII) (*F*_v_/*F*_m_), the effective photochemical yield of PSII (Φ_PSII_), and the electron transport rate (*ETR*). However, MET antagonizes the negative effects of ERY on photosynthesis, particularly at lower concentrations of the antibiotic (72h-EC_10_ and 72h-EC_25_). The exposure to ERY–MET mixtures resulted in an overall increase in photosynthetic pigments (chlorophyll *a* and carotenoids), likely reflecting a compensatory response by the alga to counterbalance impaired photosynthetic function. Nonetheless, overall pigment analysis indicated an antagonistic interaction between the two toxicants, as the magnitude of this increase was lower than predicted by the IA model. The results presented here provide new insights into the toxicological interactions between ERY and MET in microalgae and contribute to addressing the critical knowledge gap regarding the ecotoxicity of pharmaceutical–herbicide mixtures in aquatic environments.

## 1. Introduction

Herbicides are widely used in agriculture to manage weed growth and increase crop yields. On the other hand, antibiotics are essential in human and veterinary medicine for the prevention and treatment of diseases. However, the extensive use of these two classes of organic compounds has led to their release and dispersion in the environment. This is the case for the antibiotic erythromycin (ERY) and the herbicide metolachlor (MET), which have been frequently detected in aquatic systems, particularly in areas characterized by intensive agricultural and livestock activities [1,2].

ERY is a broad-spectrum macrolide antibiotic that acts by binding to the 50S ribosomal subunit of bacteria and inhibiting protein synthesis [3]. It has been frequently detected in aquatic systems in concentrations that can reach 75.5 µg/L [4].

MET is applied worldwide to crops such as corn, soybeans, potatoes, sugar beets, sunflowers, and tomatoes [5]. It is a selective and systemic herbicide from the chloroacetamide class, acting by inhibiting the synthesis of long-chain fatty acids [6]. MET’s action in plants leads to the eradication of the synthesis of chlorophyll, proteins, fatty acids, isoprenoids, and flavonoids, resulting in the impairment of cell division and plant elongation [7,8]. This herbicide has been frequently detected in surface and groundwater with concentrations exceeding 400 µg/L [9].

Ecotoxicological studies have demonstrated that herbicides and antibiotics can be toxic to aquatic non-target organisms even at environmental concentrations [10,11]. Among aquatic organisms, primary producers are the first ones to be exposed to contaminants. In addition, they constitute the base of the food chain, contributing to oxygen production, nutrient cycling, and carbon assimilation [12]. In this context, microalgae are widely used in ecotoxicity testing as they are sensitive to a large spectrum of contaminants, easy to culture and maintain in the laboratory, and representative of primary producers [13]. Within freshwater microalgae, *Raphidocelis subcapitata* is a well-established model organism, being a highly relevant bioindicator of pollutant effects [14]. For these reasons, it was the selected microorganism for the present study.

Toxicity tests of ERY and MET, separately, showed that both pollutants can exert severe effects on aquatic producer organisms. ERY inhibited growth [15,16,17] photosynthesis [18], and perturbed the defence mechanisms [19] of microalgae. This antibiotic also altered photosynthesis and induced oxidative stress in cyanobacteria [20,21]. MET has been described as a growth inhibitor in algae [22,23,24,25] and cyanobacteria [26] and as an oxidative stress promoter in algae [27].

Conventional ecotoxicological tests are typically conducted with single substances, neglecting the fact that aquatic ecosystems contain complex mixtures of chemicals. Thus, in nature, aquatic organisms are rarely, if ever, exposed, to the compounds individually, but rather to their mixtures. Moreover, the toxicity of a mixture cannot be inferred from individual chemical toxicity tests, as chemical interactions may result in additive, synergistic (higher than additive), or antagonistic (less than additive) effects [28,29,30]. Therefore, investigating the effects of toxicants as mixtures remains a major challenge, and progress in this area has been limited. This highlights the urgent need to advance with mixture risk assessment, particularly for widespread pollutants, such as ERY and MET, which may co-occur in aquatic systems. To our best knowledge, no studies have assessed the combined effects of ERY and MET on non-target organisms. Addressing this knowledge gap is essential, as risk assessments based solely on individual pollutants may underestimate the real impact of chemical mixtures on aquatic communities.

The present study aimed to assess, for the first time, the toxicity of binary mixtures of ERY and MET on a non-target organism, the freshwater microalga *R. subcapitata*. Beyond determining whether the interactions between these two contaminants result in synergistic or antagonistic outcomes, this work specifically addresses the critical lack of information on how antibiotic–herbicide mixtures affect key physiological processes in microalgae. To this end, the impact of ERY and MET mixtures on algal growth, photosynthetic activity, and photosynthetic pigment concentration was evaluated, enabling the identification of interaction-driven responses that cannot be inferred from single compound exposures. By quantifying mixture-specific impacts (such as synergistic growth inhibition, MET-mediated modulation of ERY-induced photosynthetic impairment, and antagonistic effects on pigment accumulation), this work provides mechanistic insights that can be used in ecological risk assessments associated with co-occurring pharmaceuticals and herbicides in aquatic environments.

## 2. Materials and Methods

### 2.1. Alga, Culture Medium, and Cultural Conditions

The freshwater microalga *Raphidocelis subcapitata* (strain 278/4) was obtained from Culture Collection of Algae and Protozoa (CCAP, UK). The alga was maintained on OECD medium [31] containing 2% (*w*/*v*) agar, at 4 °C.

Pre-cultures were prepared by inoculating 40 mL of OECD medium (into a 100 mL Erlenmeyer flask) with a loop of cells taken from the solid culture stored at 4 °C. All reagents comprising the culture medium were of analytical grade (PA) and were prepared in accordance with OECD guidelines [31]. Algal cells were incubated on an orbital shaker, at 100 rpm, 25 °C, for 3 days, under continuous light (8000 lux, LED lamps with a colour temperature of 4000–4200 K).

Cultures were prepared by transferring an adjusted volume of the pre-culture (up to 10 mL) to 400 mL of OECD medium in a 1 L Erlenmeyer flask, in order to obtain an initial cell concentration of 5 × 10^4^ cells/mL. Algae were incubated for 2–3 days under the cultural conditions described above.

### 2.2. Chemicals and Preparation of the Respective Solutions

The antibiotic ERY (Ref. E5389; CAS 114-07-8) and the herbicide MET (97.6%) (PESTANAL, analytical standard, Ref. 36163; CAS 51218-45-2) used in this study were provided by Sigma-Aldrich (Saint Louis, MO, USA). Stock solutions of ERY (12.6 g/L) and MET (15.2 mg/L) were prepared in dimethyl sulfoxide (DMSO; CAS 67-68-5, Sigma-Aldrich, Saint Louis, MO, USA) and deionized water, respectively. A 10 mg/L intermediate stock solution of ERY was prepared in water by dilution of ERY stock solution. Toxicity assays with ERY had a DMSO concentration <0.003%. At this DMSO concentration, the growth of *R. subcapitata* is not affected, as previously described by Machado and Soares [25].

MET is considered highly stable in aquatic systems, with hydrolysis half-lives generally above 100 days across broad pH ranges and minimal photodegradation, with reported estimates often exceeding 200 days [2,32]; modelling studies describe median half-lives of 100–300 days [33]. ERY shows half-lives varying from a few days to roughly six weeks, depending on light, pH, salinity, and matrix composition [34,35].

### 2.3. Toxicity of Individual Compounds

For single toxicity assays, the alga (1 × 10^5^ cells/mL) was exposed to eight nominal concentrations of ERY (from 2.2 to 460 µg/L) or MET (from 16 to 400 µg/L) arranged in a geometrical series, for each toxicant. EC values were determined from two independent experiments, each performed with two replicates (*n* = 4). As a control, algal cells were also grown in the absence of toxicants. After 72 h of incubation under the conditions described above (Section 2.1), the concentration of algae in the cultures was determined using an automated cell counter (TC10™, Bio-Rad, Hercules, CA, USA).

Toxicity was expressed as EC_10_, EC_25_, EC_50_, EC_75_, and EC_90_, corresponding to the concentrations of each compound that cause 10, 25, 50, 75, and 90% inhibition of algal growth, respectively, when compared with the control. The 72h-EC values for ERY and MET were calculated by linear interpolation using the software TOXCALC version 5.0.32 (Tidepool Scientific Software, McKinleyville, CA, USA).

### 2.4. Toxicity of Binary Mixtures of ERY and MET

For the mixture assays, nine combinations of the two toxicants were established based on the individual 72h-EC_10_, 72h-EC_25_, and 72h-EC_50_ values. These EC values for each substance were selected to construct mixtures spanning low to moderate biological stress. This selection follows established mixture-toxicity methodology, where EC values act as biologically relevant reference points for comparing compounds and assessing interactions across effect levels [36].

Mixtures were prepared in 250 mL Erlenmeyer flasks by adding OECD medium and the appropriate concentration of each toxicant according to the experimental design. Flasks were subsequently inoculated with the alga (1 × 10^5^ cells/mL), adjusted to a final volume of 100 mL, and incubated for 72 h under the conditions described above (Section 2.1). Control assays consisted of algal cultures without toxicants, as well as algae exposed individually to 72h-EC_10_, 72h-EC_25_, and 72h-EC_50_ concentrations of ERY and MET. Mixtures and controls were prepared in triplicate (*n* = 3).

After a 72 h incubation period, the effects on microalgal growth, photosynthetic performance and pigment content were assessed. Algal concentration was quantified as described in Section 2.3; photosynthetic performance and pigments content were evaluated as detailed in Section 2.5 and Section 2.6, respectively.

### 2.5. Evaluation of Photosynthetic Performance

Algal photosynthetic performance was measured by Pulse Amplitude Modulated (PAM) fluorometry (Junior PAM, Walz, Germany). To this end, the algal suspensions (3 × 10^6^ cells/mL) were dark-adapted for 30 min before measurement. Minimum (*F*_0_) and maximum (*F*_m_) fluorescence yields were recorded to calculate the maximum photochemical quantum yield of photosystem II (*F*_v_/*F*_m_), also known as photosystem II (PSII) activity [37]. To evaluate light energy utilization, continuous actinic light (190 µmol photons m^−2^ s^−1^) was applied with saturating pulses every 20 s for 5 min. During this period, minimum and maximum fluorescence under light (*F*′_0_ and *F*′_m_) were measured. The *F*_v_/*F*_m_, the effective photochemical yield of PSII (Φ_PSII_), and electron transport rate through PSII (*ETR*) were calculated according to the standard equations and mathematical formulations [38,39,40], using WinControl software (v3.2.2). In each biological sample, PAM measurements were performed on ten technical replicates.

### 2.6. Quantification of Pigments Content

For the quantification of photosynthetic pigments, 5.0 mL of algal suspension (3 × 10^6^ cells/mL) was harvested by centrifugation at 2500× *g* for 10 min. The supernatant was discarded, and the resulting cell pellet was resuspended in 5.0 mL of 90% (*v*/*v*) acetone (VWR Chemicals, Rosny-sous-Bois, France) for pigment extraction. Samples were placed at 4 °C, in the dark, for 20 h, to ensure complete extraction of pigments. Subsequently, samples were centrifuged at 2500× *g* for 10 min, and the clarified supernatant was collected for analysis. The absorbance of the supernatant was measured at 630, 647, 664, and 750 nm to determine chlorophyll *a* (Chl *a*) concentration according to the equations described by APHA [41]. Carotenoids (CAR) content was quantified based on absorbance at 480 nm, following the equation described by Strickland and Parsons [42]. In each biological sample, pigment determinations were performed on two technical replicates.

### 2.7. Assessment of Mixture Interactions

The observed effect resulting from the joint toxicity of chemical mixtures may be larger (synergism) or smaller (antagonism) than predicted by specific models, such as concentration addition (CA) or independent action (IA). The IA model has been applied to describe mixtures composed of compounds with dissimilar modes of action, affecting the organism at different target sites or biological pathways [36].

Based on the IA model, Gottardi et al. [43] proposed equations to calculate the predicted and observed effects, as well as the extent of synergism among compounds in a mixture. Thus, the interaction between ERY and MET was assessed using the mathematical framework proposed by Gottardi et al. [43], considering growth, photosynthetic performance, and pigment content as endpoints.

The predicted effect of the mixtures in microalgae can be calculated using the equation:

(1) $$Predicted\;effect=\frac{Toxic\;1\;effect}{Control}\times \frac{Toxic\;2\;effect}{Control}$$

where Toxic 1 effect and Toxic 2 effect correspond to the values obtained for the biological parameter in microalga *R. subcapitata* individually exposed to toxicants 1 and 2, respectively. Control represents the value of the same biological parameter in the microalga that was not exposed to the toxicants.

On the other hand, the observed effect of the mixture on the microalga can be calculated using the equation:

(2) $$Observed\;effect=\frac{Mixture\;effect}{Control}$$

where mixture effect represents the value of the biological parameter in microalga exposed to the mixture.

To determine the extent of synergism or antagonism, the synergy ratio (SR) is calculated using the following equation:

(3) $$Synergy\;ratio=\frac{Predicted\;effect}{Observed\;effect}$$

A SR greater than 1 indicates synergism, whereas a value below 1 indicates antagonism.

### 2.8. Reproducibility of the Results and Data Analysis

In the determination of ECs values of the individual toxicants, results are presented as the mean ± standard deviation (SD) of two independent experiments performed in duplicate (*n* = 4). For the toxicity assessment of the compound mixtures, data are presented as mean ± SD of an experiment performed in triplicate (*n* = 3).

For each ERY–MET mixture and each evaluated parameter, one-way ANOVA was performed comparing the control, the corresponding individual ERY concentration, the corresponding individual MET concentration, and the respective ERY–MET mixture. Differences between experimental groups were considered statistically significant at *p* < 0.05. When significant differences were detected by ANOVA, Tukey–Kramer post hoc tests were used for pairwise comparisons. The corresponding *F*-values and *p*-values from the one-way ANOVA analyses are presented in Table S1 (Supplementary Material).

## 3. Results

### 3.1. Individual and Combined Effects of ERY and MET on the Microalga Growth

As a first approach, the toxicity of ERY and MET toward *R. subcapitata* was assessed through dose–response curves (Figure S1, Supplementary Material). Cell growth inhibition was used as the endpoint to determine the respective 72h-EC values. It was found that ERY and MET exhibited 72h-EC_50_ values of 35 and 44 µg/L, respectively (Table S2, Supplementary Material).

To characterize ERY and MET interactions, at environmentally relevant concentrations, the microalga *R. subcapitata* was exposed to nine mixtures derived from combinations of the EC_10_, EC_25_, and EC_50_ values of each toxicant. For comparative purposes, the individual effect of each antibiotic and herbicide concentration was also assessed. Table S3 (Supplementary Material), summarizes several examples of ERY and MET concentrations reported in the literature for natural surface waters and groundwaters [4,9,44,45,46,47,48,49,50].

**Figure 1:**
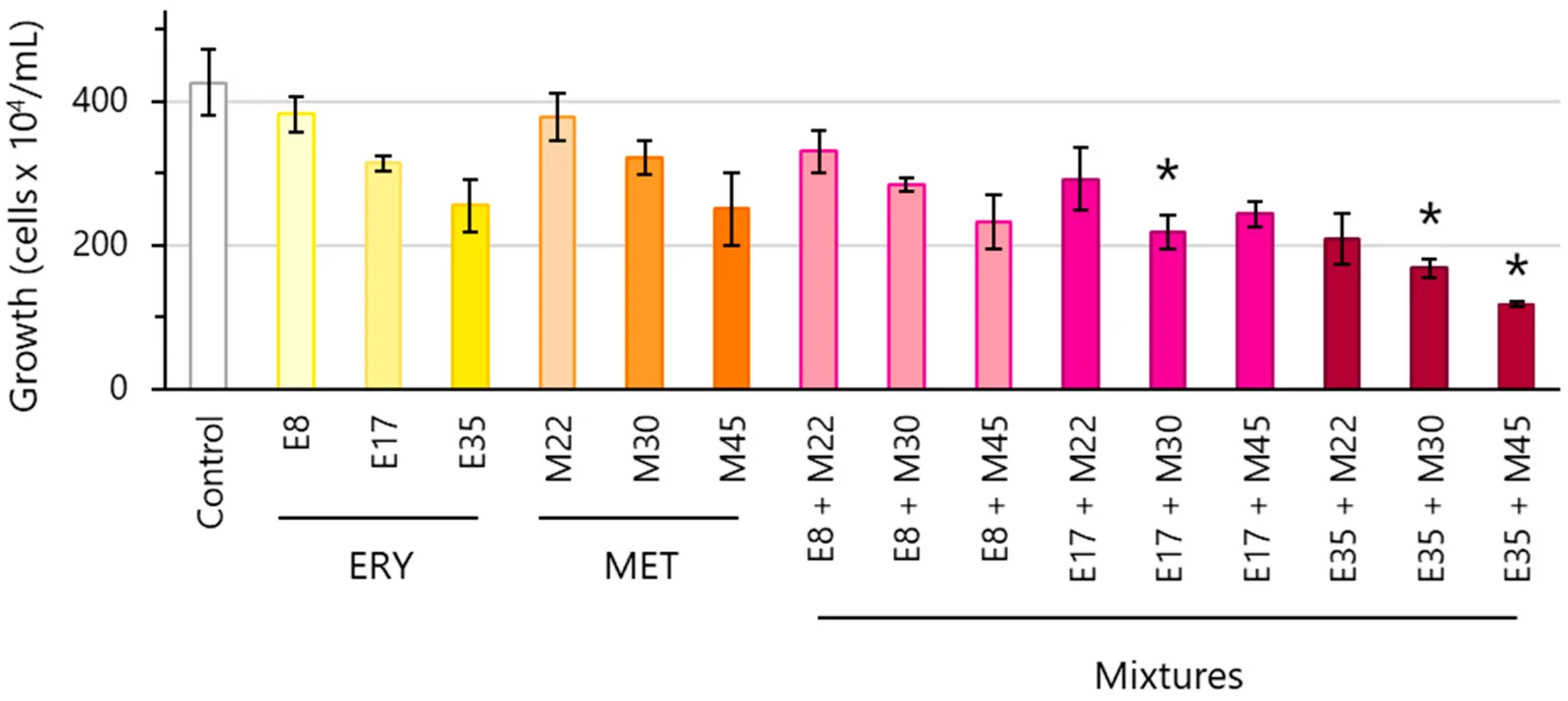
Impact of erythromycin (ERY) and metolachlor (MET), individually and in combination, on the growth of *R. subcapitata*. Algae were inoculated in OECD medium in the absence (control) or presence of ERY, MET, or their mixtures. Numbers following the labels E (erythromycin) and M (metolachlor) indicate the concentration (µg/L) of each toxicant. Error bars represent the standard deviation. Asterisks (*) denote significant differences (*p* < 0.05) in growth compared to both the control and respective individual toxicant concentrations. Statistical significance was determined via one-way ANOVA followed by the Tukey–Kramer post hoc test.

The evaluation of the impact of ERY–MET mixtures on microalga, over 72 h, revealed that growth was progressively inhibited as toxicant concentrations increased (Figure 1).

Statistical analysis of the effect of the mixtures of pollutants showed that exposure to E17 + M30, E35 + M30, and E35 + M45 resulted in a significant reduction in growth (*p* < 0.05; ANOVA) compared with the control (algae not exposed to toxicants) and with the corresponding individual concentrations of ERY and MET (Figure 1).

The possible synergistic or antagonistic interactions of the three ERY–MET mixtures on algal growth inhibition (a sub-lethal endpoint) were assessed. For this purpose, the observed effect was compared with the predicted effect from exposure to the mixture of single compounds, using the synergism ratio, as described by Gottardi et al. [43].

The analysis was conducted considering the independent action (IA) model, assuming that ERY and MET act independently on a common biological endpoint, through distinct mechanisms of action, thereby affecting the algae via separate targets or metabolic pathways [36]. For the E17 + M30, E35 + M30, and E35 + M45 mixtures, synergy ratios of 1.1, 1.2, and 1.3, respectively, were calculated (Table 1), indicating a synergistic interaction [36]. Hence, these ERY–MET mixtures produced a negative effect (algal growth inhibition) that was 9%, 13%, and 20% higher than predicted, respectively, based on the individual toxicities of the compounds.

Given the fundamental role of photosynthesis in microalgal physiology, the toxicological impact of ERY–MET binary mixtures was further investigated by evaluating their effects on photosynthetic activity and pigment content, as complementary endpoints to growth inhibition.

### 3.2. Disturbances in Photosynthetic Performance Induced by Individual Toxicants and Their Binary Mixtures

**Figure 2:**
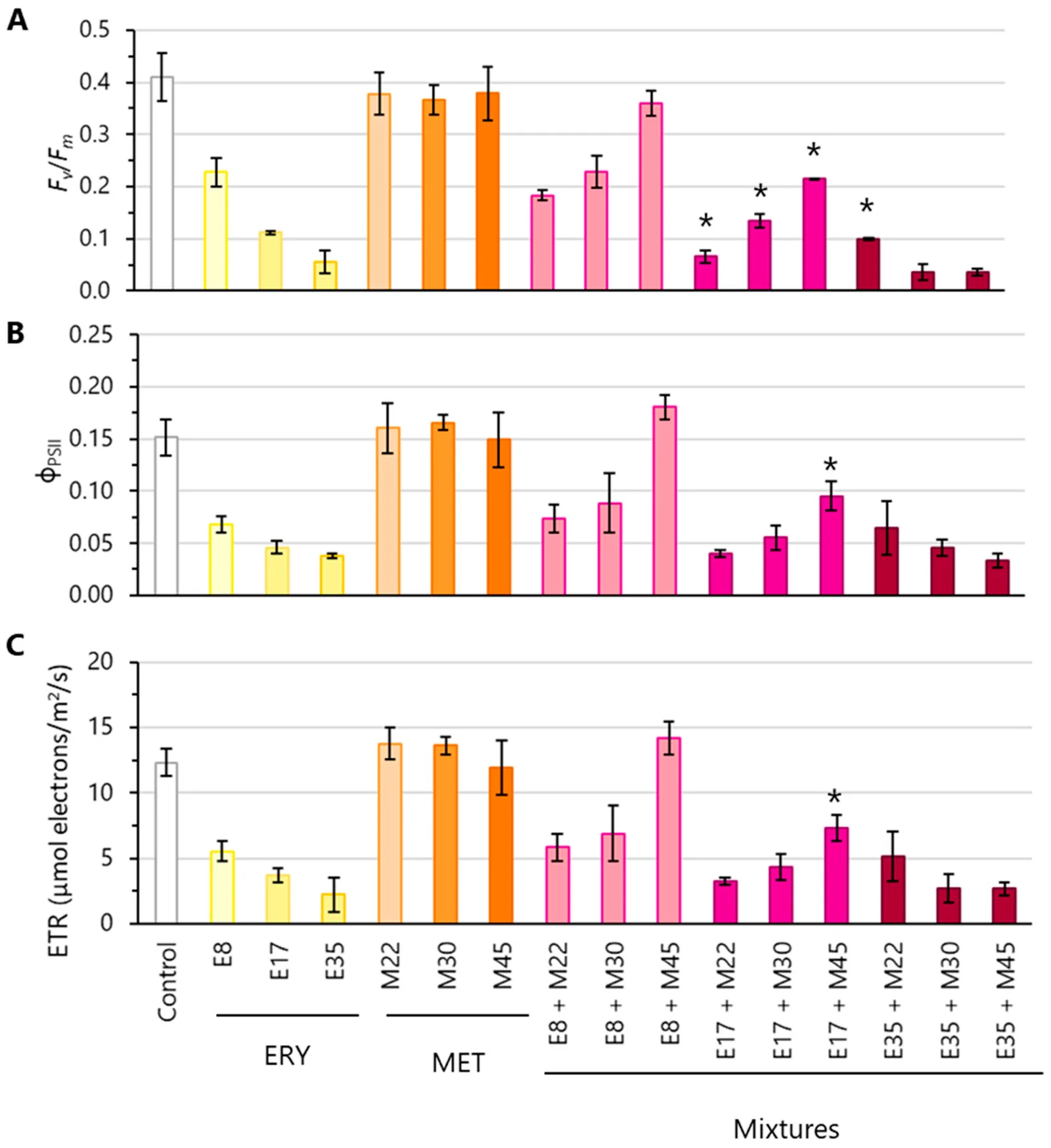
Influence of ERY, MET, and their mixtures on the photosynthetic performance of *R. subcapitata*. (**A**) maximum photochemical yield of PSII (*F*_v_/*F*_m_); (**B**) effective photochemical yield of PSII (Φ_PSII_); (**C**) electron transport rate (*ETR*). X-axis abbreviations are as defined in Figure 1. Error bars represent the standard deviation. Asterisks (*) denote significant differences (*p* < 0.05) in the photosynthetic parameter compared to both the control and respective individual toxicant concentrations. Statistical significance was determined via one-way ANOVA followed by the Tukey–Kramer post hoc test.

When tested individually, ERY at concentrations ranging from 8 to 35 µg/L exerted a pronounced inhibitory effect on algal photosynthesis, reducing the maximum photochemical yield of photosystem II (PSII) (*F*_v_/*F*_m_) by 45–87%. In contrast, exposure of *R. subcapitata* to MET within the concentration range from 22 to 45 µg/L did not produce a pronounced alteration of *F*_v_/*F*_m_ (Figure 2A), suggesting that MET, at these concentrations, has no, or only a slight, impact on the photosynthetic activity of the alga.

The results from exposing the algae to the different ERY–MET mixtures showed that, except for E8 + M45, all combinations impaired algal photosynthetic capacity, as evidenced by consistent alterations across all evaluated photosynthetic parameters (Figure 2). Regarding the *F*_v_/*F*_m_ ratio, exposure to ERY–MET mixtures induced distinct patterns of change in the microalgae. The combinations E8 + M22 and E17 + M22 induced a greater reduction in photosynthetic activity (*F*_v_/*F*_m_) compared to the respective individual concentrations of ERY. In turn, the mixtures E8 + M30, E35 + M30, and E35 + M45 induced a reduction of *F*_v_/*F*_m_ similar to that caused by ERY alone. In contrast, the reduction in the *F*_v_/*F*_m_ ratio for the E8 + M45, E17 + M30, and E35 + M22 mixture was less pronounced than that induced by the respective individual ERY concentrations. Notably, in the case of E8 + M45, the inhibitory effect of ERY on *F*_v_/*F*_m_ was completely suppressed by MET (Figure 2A).

Statistical treatment of the results revealed that the mixtures E17 + M22, E17 + M30, E17 + M45, and E35 + M22 disturbed algal photosynthesis (*F*_v_/*F*_m_) significantly more than both the control and the corresponding individual toxicant concentrations (Figure 2A). The SR for the mixture E17 + M22 was 1.6, indicating a synergistic interaction (Table 1). Accordingly, this mixture produced a reduction in the *F*_v_/*F*_m_ ratio 36% higher than predicted based on the individual toxicity of the compounds. In contrast, the SR for mixtures E17 + M30, E17 + M45, and E35 + M22 were 0.73, 0.47, and 0.50, respectively, suggesting an antagonistic effect between the toxicants (Table 1); these mixtures induced a reduction in the *F*_v_/*F*_m_ ratio that was 38%, 112%, and 100%, respectively, lower than predicted from the individual effects.

The individual effects of ERY and MET on the effective photochemical yield of PSII (Φ_PSII_), and the electron transport rate (*ETR*) of the algae followed a similar trend to that observed with the *F*_v_/*F*_m_ ratio (Figure 2B,C). Specifically, ERY caused a progressive and substantial reduction in Φ_PSII_ (between 55 and 75%) and *ETR* (between 55 and 82%). As it occurred with the *F*_v_/*F*_m_ ratio, MET, within the tested concentration range, did not cause a meaningful alteration in these parameters (Figure 2B,C).

The effect of the mixture E17 + M45 on Φ_PSII_, and *ETR*, differed significantly from both the control and the corresponding individual concentrations of the toxicants (Figure 2B,C). This mixture exhibited synergy ratios of 0.48 and 0.49 for Φ_PSII_ and *ETR*, respectively (Table 1), indicating an antagonistic interaction between the toxicants relative to these photosynthetic parameters, which corresponded to values 110% and 103%, respectively, lower than predicted from the individual effects.

### 3.3. Impact of ERY, MET, and Their Mixtures on Microalgae Photosynthetic Pigments

**Figure 3:**
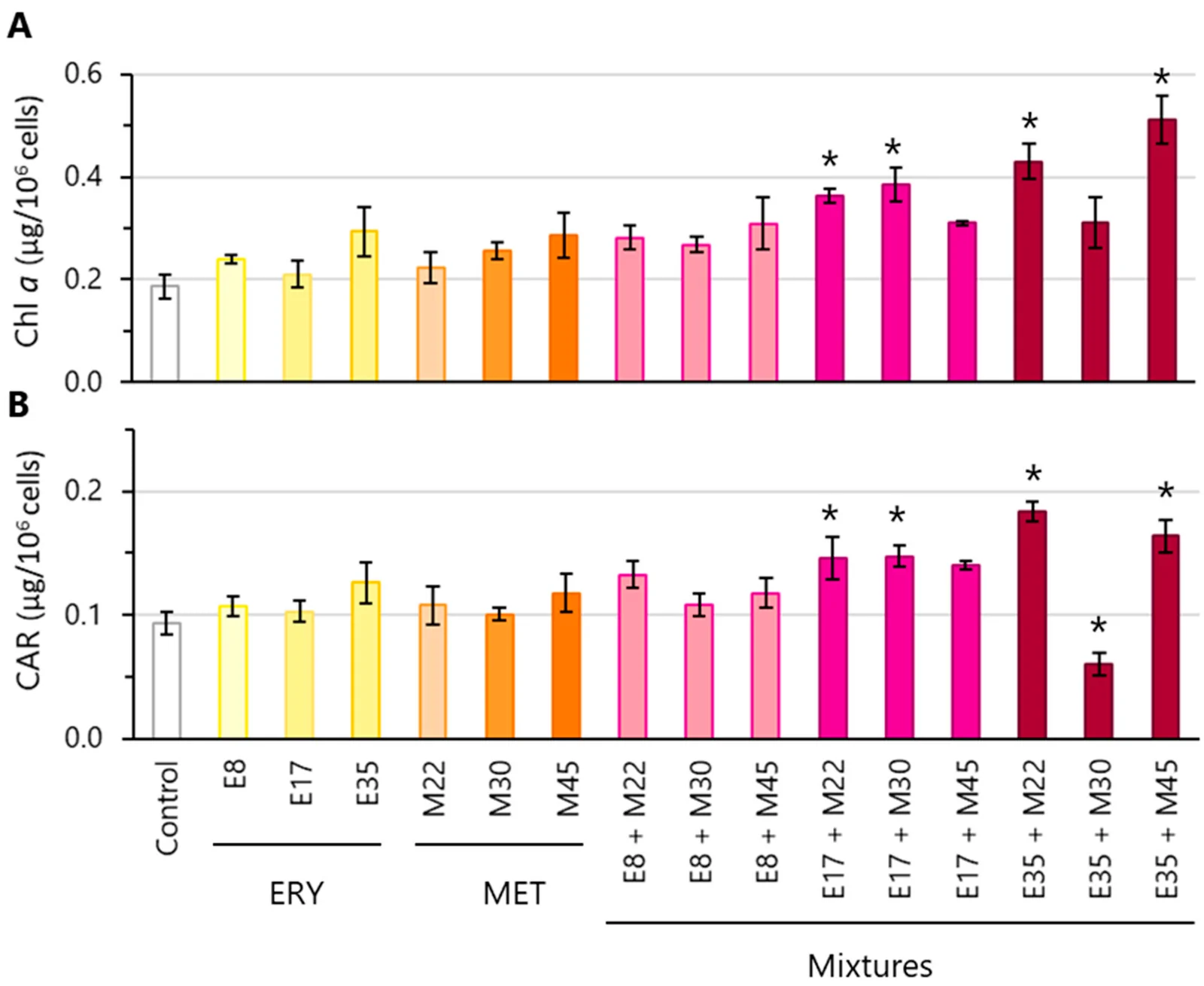
Effect of ERY, MET and their mixtures on the photosynthetic pigments of *R. subcapitata*. (**A**) chlorophyll *a* (Chl *a*) content; (**B**) carotenoids (CAR) content. See Figure 1 for x-axis abbreviation definitions. Error bars represent the standard deviation. Asterisks (*) denote significant differences (*p* < 0.05) in the pigments content compared to both the control and respective individual toxicant concentrations. Statistical significance was determined via one-way ANOVA followed by the Tukey–Kramer post hoc test.

No effect, or a slight increase, ranging from 28 to 57% and 19 to 53%, in chlorophyll *a* (Chl *a*) content was observed in algae exposed to ERY and MET, respectively (Figure 3A).

All tested mixtures of ERY + MET induced an increase in the Chl *a* content of the alga. The mixtures E17 + M22, E17 + M30, E35 + M22, and E35 + M45 induced a significantly higher Chl *a* levels compared to both the control and the individual exposures to each toxicant (Figure 3A). The calculated synergistic ratios for these mixtures were 0.74, 0.71, 0.83, and 0.89, respectively (Table 1), indicating an antagonistic interaction between ERY and MET, which corresponded to Chl *a* values 36%, 40%, 21%, and 13%, respectively, lower than predicted from the individual effects.

A similar pattern was observed for carotenoids (CAR), both under individual exposures and in mixtures. Thus, no effect or a marginal effect on CAR content was detected in algae exposed individually to ERY or MET, respectively (Figure 3B).

The mixtures, E17 + M22, E17 + M30, E35 + M22, E35 + M30, and E35 + M45 yield CAR levels that differed significantly from those of the control and individual toxicant concentrations. Except for E35 + M30, these mixtures induced an increase in algal CAR content (Figure 3B). The calculated synergy ratios (0.75 to 0.94) denoted an antagonistic interaction between ERY and MET, resulting in CAR levels 6 to 25% lower than those predicted from the individual effects.

## 4. Discussion

ERY and MET are considered contaminants of emerging concern (CECs) due to their expanding use, environmental persistence, increasing environmental detection, and toxicity even at low concentrations [51,52]. These two compounds presented low (<50 µ/L) 72h-EC_50_ values, which can be classified as very toxic (≤1 mg/L) to the aquatic environment [53,54].

Although ERY and MET ultimately lead to the same effect (growth inhibition), their modes of action (MoA) in *R. subcapitata* appear to involve different pathways, given the distinct adverse effects they induce. In this alga, ERY induces the down-regulation of genes associated with porphyrin and chlorophyll metabolism pathways [55], reduces photosynthetic activity [17,18], and affects mitochondrial function without inducing reactive oxygen species (ROS) production or altering reduced glutathione (GSH) levels [17]. In contrast, MET induces a marked increase in cell size [56,57], promotes the formation of a palmelloid-like phenotype [58], and triggers ROS accumulation with a deep impact on the algal redox defence machinery [27], without affecting (or affecting marginally) photosynthetic activity. Taken together, these data strongly support the adoption of the independent action (IA) model, used in this work, in the analysis of mixtures composed of ERY and MET.

Overall, increasing the individual concentrations of toxicants within the binary mixtures led to greater inhibition of algal growth. In all mixtures that showed significantly different growth compared to the control and the individual treatments of ERY or MET, a synergistic effect between the two substances was found. A similar effect was reported for mixtures of macrolide antibiotics (erythromycin and clarithromycin) with the azole fungicide ketoconazole, using growth inhibition of *R. subcapitata* as the endpoint [59].

Regarding the combined effects of both compounds on algal photosynthetic performance, an overall reduction in PSII efficiency was observed. Interestingly, MET seems to antagonize the negative impact of ERY. Consistently, the E17 + M45 mixture elicited an antagonistic response across all photosynthetic parameters analyzed. An antagonistic interaction was also observed for MET, which mitigated the toxic action of ERY on photosynthetic performance (*F*_v_/*F*_m_) for the E17 + M30 and E35 + M22 mixtures. The partial mitigation of ERY toxicity by MET suggests that the two toxicants may interfere with distinct but interacting stress-response pathways, leading to compensatory mechanisms that attenuate ERY-induced toxicity. These mechanisms may involve the activation of antioxidant defences, stimulation of detoxification routes, or adjustments in cellular metabolism that support PSII repair and pigment synthesis. Previous studies have reported that binary and multi-component mixtures of antibacterial agents can exert antagonistic effects on *R. subcapitata* [28,60]. Antagonistic interactions have also been reported for macrolide antibiotic mixtures (erythromycin and roxithromycin) in the microalga *Chlorella pyrenoidosa* [61]. Transcriptomic analysis revealed that co-exposure to toxicants triggered the upregulation of key xenobiotic detoxification genes, namely cytochrome P450, glutathione S-transferase (GST), and ABC transporter systems in *C. pyrenoidosa*, which actively accelerate the metabolism and cellular efflux of the toxicants. Such mechanisms may similarly contribute to the reduced photosynthetic impairment observed in ERY-MET mixtures.

It was observed that both ERY and MET induced a slight increase in Chl *a* and CAR, a pattern consistent with previous studies reporting photosynthetic pigment upregulation in microalgae (including *R. subcapitata*) experiencing moderate stress and displaying a reduced PSII efficiency, due to the exposure to herbicides [62,63,64] or high salinity [65]. Given the essential role of Chl *a* in light harvesting [66] and the photoprotective and antioxidant functions of CAR [67], the photosynthetic pigment upregulation can be seen as a physiological adjustment (compensatory response) in microalgae exposed to moderate stress. In this regard, different authors proposed that the Chl *a* upregulation, aimed to maximize light harvesting, could be an adaptive strategy to compensate for the reduction in photosynthetic efficiency under adverse conditions [63,64,68]. Similarly, the increase in CAR can be interpreted as a mechanism to dissipate excess energy, remove ROS, and stabilize pigment–protein complexes when, under stress, there is a decrease in the activity/efficiency of PSII [69,70].

When ERY and MET were combined, globally, photosynthetic pigments also increased, indicating that the alga continued to activate this compensatory mechanism. However, the magnitude of pigment upregulation was 13–40% lower than predicted by the independent action model. Therefore, in this context, antagonism corresponds to a reduced compensatory response compared to what is expected from individual effects [36]. Two hypotheses are possible to explain this antagonism. The first possibility corresponds to a reduced algal compensation. Thus, under the stress imposed by the mixtures, the compensatory pigment response was lower than predicted from single-compound exposures, indicating a reduced capacity of the algae to establish an effective defensive mechanism. This possibility is consistent with the observed growth inhibition and photosynthetic parameters reduction (*F_v_/F_m_*, Φ_PSII_, *ETR*), which indicates that the alga remained under functional stress despite the increase in photosynthetic pigment accumulation. The second possibility is that, theoretically, the mixtures exerted lower effective stress than predicted. This possibility cannot be excluded, given that MET partially mitigates ERY-induced impairment of PSII (particularly at lower ERY concentrations). Under this scenario, less pigment upregulation would be required to maintain photosynthetic function. Nevertheless, this hypothesis appears less likely, as photosynthetic performance was not restored to control levels, indicating that the algae remained under stress.

The observed growth reduction and disturbances in photosynthetic efficiency in *R. subcapitata* exposed to ERY-MET mixtures may have broad ecological implications. It is known that phytoplankton accounts for more than fifty percent of the net primary production on Earth [71]. On the other hand, as component of phytoplankton, microalgae are at the base of the food web (primary producers) [72]. Thus, even a moderate decline in microalgal growth could weaken the food chain with potential cascading effects throughout the ecosystem. In addition, alterations in pigment composition and impaired PSII function (affecting microalgal photosynthesis), can also influence the efficiency of carbon sequestration, as photosynthesis is the most dominant carbon fixation pathway [73,74,75]. Furthermore, changes in microalgal photosynthetic performance can also affect water quality by modifying oxygen dynamics, thus altering redox conditions and nutrient cycling [12,76]. Therefore, the combined effects of ERY and MET, here observed, may extend beyond specific cellular responses, potentially influencing ecosystem functioning in freshwater environments contaminated with these pollutants.

This study was conducted using a well-known and ecologically relevant freshwater microalga (*R. subcapitata*) with a short-term (72 h) exposure, which provides a controlled and sensitive framework for detecting early physiological responses but does not capture longer-term ecological dynamics. All values of the toxicants (single and in mixtures) correspond to nominal concentrations. For a more complete characterization of the environmental risk associated with the mixture of toxicants studied here (ERY and MET), long-term studies and assessments of possible effects across additional trophic levels will be required.

To conclude, the mixtures composed of ERY and MET, at low and environmentally relevant concentrations, synergistically inhibited algal growth. In addition, the mixtures of the two contaminants had a profound impact on photosynthetic performance, as reflected by reductions in *F*_v_/*F*_m_, Φ_PSII_, and *ETR*. Interestingly, MET antagonized the detrimental effects of ERY on photosynthesis, particularly at low concentrations of the antibiotic. Conversely, exposure to ERY–MET mixtures resulted in an overall increase in photosynthetic pigments (Chl *a* and CAR), likely reflecting a compensatory mechanism activated by the alga to mitigate the decline in photosynthetic efficiency. Despite the increase in pigment accumulation, the interaction between the two toxicants remained predominantly antagonistic.

Taken together, the results presented here uncover previously unreported physiological responses of *R. subcapitata* to antibiotic–herbicide mixtures, highlighting the complexity of combined toxicant effects in aquatic primary producers. By elucidating both synergistic and antagonistic interactions across key physiological endpoints (growth, photosynthetic performance, and photosynthetic pigments concentrations), this study contributes new knowledge to the ecotoxicological assessment of pharmaceutical–herbicide mixtures and reinforces the need to consider the mixture effects in the risk assessment of these compounds for aquatic ecosystems.

## Figures and Tables

**Table 1:** Evaluation of antagonistic or synergistic interactions in mixtures of erythromycin and metolachlor on the physiology of *R. subcapitata*.

Parameter	Mixture	Predicted Effect	Observed Effect	Synergy Ratio	Interaction
Growth	**E17 + M30**	0.56	0.51	**1.1**	**S**
	**E35 + M30**	0.45	0.39	**1.2**	**S**
	**E35 + M45**	0.35	0.28	**1.3**	**S**
*F*_v_/*F*_m_	**E17 + M22**	0.25	0.16	**1.6**	**S**
	E17 + M30	0.24	0.33	0.73	A
	E17 + M45	0.25	0.53	0.47	A
	E35 + M22	0.12	0.24	0.50	A
Φ_PSII_	E17 + M45	0.30	0.63	0.48	A
*ETR*	E17 + M45	0.29	0.59	0.49	A
Chl *a*	E17 + M22	1.4	1.9	0.74	A
	E17 + M30	1.5	2.1	0.71	A
	E35 + M22	1.9	2.3	0.83	A
	E35 + M45	2.4	2.7	0.89	A
CAR	E17 + M22	1.3	1.6	0.81	A
	E17 + M30	1.2	1.6	0.75	A
	E35 + M22	1.6	2.0	0.80	A
	**E35 + M30**	1.5	0.65	**2.3**	**S**
	E35 + M45	1.7	1.8	0.94	A

Synergy ratio > 1: synergism (S); synergy ratio < 1: antagonism (A). Mixture abbreviations are as defined in Figure 1. CAR—carotenoids; Chl *a*—chlorophyll *a*; *ETR*—electron transport rate; *F*_v_/*F*_m_—maximum photochemical yield of photosystem II (PSII); Φ_PSII_—effective photochemical yield of PSII.

## Data Availability

The original contributions presented in this study are included in the article/Supplementary Material. Further inquiries can be directed to the corresponding authors.

## Supplementary Materials

The following supporting information can be downloaded at: https://www.mdpi.com/article/10.3390/toxics14080645/s1, Figure S1: Dose–response curves of *R. subcapitata* exposed to erythromycin (ERY) or metolachlor (MET); Table S1: One-way ANOVA results (*F*-values and *p*-values) for the effects of ERY-MET mixtures on the physiological parameters of *R. subcapitata*; Table S2: Effect concentrations (EC) of ERY and MET on *R. subcapitata*; Table S3: Environmental concentrations of ERY and MET in natural waters. References [4,9,44,45,46,47,48,49,50] cite in Supplementary Materials.

## References

[1] Fernandes J.P., Almeida C.M.R., Salgado M.A., Carvalho M.F., Mucha A.P. (2021). Pharmaceutical Compounds in Aquatic Environments-Occurrence, Fate and Bioremediation Prospective. Toxics.

[2] Rivard L. (2003). Environmental Fate of Metolachlor.

[3] Smith A.D., Datta S.P., Smith G.H., Campbell P.N., Bentley R., McKenzie M.H. (2000). Oxford Dictionary of Biochemistry and Molecular Biology.

[4] Schafhauser B.H., Kristofco L.A., de Oliveira C.M.R., Brooks B.W. (2018). Global Review and Analysis of Erythromycin in the Environment: Occurrence, Bioaccumulation and Antibiotic Resistance Hazards. Environ. Pollut..

[5] Rachuy J.S., Fennimore S.A. (2022). Evaluation of Sulfentrazone and S-Metolachlor in *Brassica* Vegetables. Weed Technol..

[6] Gotz T., Böger P. (2004). The Very-Long-Chain Fatty Acid Synthase Is Inhibited by Chloroacetamides. Z. Naturforschung C.

[7] Deal L.M., Hess F.D. (1980). An Analysis of the Growth Inhibitory Characteristics of Alachlor and Metolachlor. Weed Sci..

[8] Singh B., Singh K. (2016). Microbial Degradation of Herbicides. Crit. Rev. Microbiol..

[9] U.S. Environmental Protection Agency (2019). US-EPA Draft Ecological Risk Assessment for Registration Review for Metolachlor/S-Metolachlor: EPA–HQ–OPP–2014–0772.

[10] Wan N.-F., Fu L., Dainese M., Kiær L.P., Hu Y.-Q., Xin F., Goulson D., Woodcock B.A., Vanbergen A.J., Spurgeon D.J. (2025). Pesticides Have Negative Effects on Non-Target Organisms. Nat. Commun..

[11] Yang Q., Gao Y., Ke J., Show P.L., Ge Y., Liu Y., Guo R., Chen J. (2021). Antibiotics: An Overview on the Environmental Occurrence, Toxicity, Degradation, and Removal Methods. Bioengineered.

[12] Booth J.M., Giomi F., Daffonchio D., McQuaid C.D., Fusi M. (2023). Disturbance of Primary Producer Communities Disrupts the Thermal Limits of the Associated Aquatic Fauna. Sci. Total Environ..

[13] Lee J., Hong S., An S.-A., Khim J.S. (2023). Methodological Advances and Future Directions of Microalgal Bioassays for Evaluation of Potential Toxicity in Environmental Samples: A Review. Environ. Int..

[14] Machado M.D., Soares E. (2024). V Features of the Microalga *Raphidocelis subcapitata*: Physiology and Applications. Appl. Microbiol. Biotechnol..

[15] Eguchi K., Nagase H., Ozawa M., Endoh Y.S., Goto K., Hirata K., Miyamoto K., Yoshimura H. (2004). Evaluation of Antimicrobial Agents for Veterinary Use in the Ecotoxicity Test Using Microalgae. Chemosphere.

[16] Isidori M., Lavorgna M., Nardelli A., Pascarella L., Parrella A. (2005). Toxic and Genotoxic Evaluation of Six Antibiotics on Non-Target Organisms. Sci. Total Environ..

[17] Machado M.D., Soares E.V. (2019). Impact of Erythromycin on a Non-Target Organism: Cellular Effects on the Freshwater Microalga *Pseudokirchneriella subcapitata*. Aquat. Toxicol..

[18] Liu B., Nie X., Liu W., Snoeijs P., Guan C., Tsui M.T.K. (2011). Toxic Effects of Erythromycin, Ciprofloxacin and Sulfamethoxazole on Photosynthetic Apparatus in *Selenastrum capricornutum*. Ecotoxicol. Environ. Saf..

[19] Nie X.P., Liu B.Y., Yu H.J., Liu W.Q., Yang Y.F. (2013). Toxic Effects of Erythromycin, Ciprofloxacin and Sulfamethoxazole Exposure to the Antioxidant System in *Pseudokirchneriella subcapitata*. Environ. Pollut..

[20] Wan J., Guo P., Peng X., Wen K. (2015). Effect of Erythromycin Exposure on the Growth, Antioxidant System and Photosynthesis of *Microcystis flos-aquae*. J. Hazard. Mater..

[21] Deng C.N., Zhang D.Y., Pan X.L. (2014). Toxic Effects of Erythromycin on Photosystem I and II in *Microcystis aeruginosa*. Photosynthetica.

[22] Ebenezer V., Ki J.S. (2013). Quantification of Toxic Effects of the Herbicide Metolachlor on Marine Microalgae *Ditylum brightwellii* (Bacillariophyceae), *Prorocentrum minimum* (Dinophyceae), and *Tetraselmis suecica* (Chlorophyceae). J. Microbiol..

[23] Thakkar M., Randhawa V., Wei L. (2013). Comparative Responses of Two Species of Marine Phytoplankton to Metolachlor Exposure. Aquat. Toxicol..

[24] Vallotton N., Moser D., Eggen R.I.L., Junghans M., Chèvre N. (2008). S-Metolachlor Pulse Exposure on the Alga *Scenedesmus vacuolatus*: Effects during Exposure and the Subsequent Recovery. Chemosphere.

[25] Machado M.D., Soares E.V. (2019). Sensitivity of Freshwater and Marine Green Algae to Three Compounds of Emerging Concern. J. Appl. Phycol..

[26] Chen S., Zhang L., Chen H., Chen Z., Wen Y. (2019). Enantioselective Toxicity of Chiral Herbicide Metolachlor to *Microcystis aeruginosa*. J. Agric. Food Chem..

[27] Machado M.D., Soares E. (2021). V Exposure of the Alga *Pseudokirchneriella subcapitata* to Environmentally Relevant Concentrations of the Herbicide Metolachlor: Impact on the Redox Homeostasis. Ecotoxicol. Environ. Saf..

[28] González-Pleiter M., Gonzalo S., Rodea-Palomares I., Leganes F., Rosal R., Boltes K., Marco E., Fernandez-Pinas F. (2013). Toxicity of Five Antibiotics and Their Mixtures towards Photosynthetic Aquatic Organisms: Implications for Environmental Risk Assessment. Water Res..

[29] Pérez J., Domingues I., Soares A.M.V.M., Loureiro S. (2011). Growth Rate of *Pseudokirchneriella subcapitata* Exposed to Herbicides Found in Surface Waters in the Alqueva Reservoir (Portugal): A Bottom-up Approach Using Binary Mixtures. Ecotoxicology.

[30] Barata C., Baird D.J., Nogueira A.J.A., Soares A.M.V.M., Riva M.C. (2006). Toxicity of Binary Mixtures of Metals and Pyrethroid Insecticides to *Daphnia magna* Straus. Implications for Multi-Substance Risks Assessment. Aquat. Toxicol..

[31] OECD (2011). Test No. 201: Freshwater Alga and Cyanobacteria, Growth Inhibition Test.

[32] LeBaron H.M., McFarland J., Simoneaux B.J., Ebert E., Kearney P.C., Kaufman D.D. (1988). Metolachlor. Herbicides: Chemistry, Degradation and Mode of Action.

[33] Waters D.K., Silburn D.M. (2023). Calculating Pesticide Half-Lives in Reservoirs for Model Parameterization. Proceedings of the Proceedings of the 25th International Congress on Modelling and Simulation (MODSIM2023), 9–14 July 2023.

[34] Wu Y., Yang C., Tang T., Guo X., Dang Z. (2015). Distribution of Erythromycin in Aquatic Ecosystem: Microcosm Study. Huanjing Kexue Xuebao/Acta Sci. Circumst..

[35] Batchu S.R., Panditi V.R., O’Shea K.E., Gardinali P.R. (2014). Photodegradation of Antibiotics under Simulated Solar Radiation: Implications for Their Environmental Fate. Sci. Total Environ..

[36] Cedergreen N., Svendsen C., Backhaus T. (2012). Toxicity Prediction of Chemical Mixtures. Encyclopedia of Environmental Management.

[37] Sipka G., Magyar M., Mezzetti A., Akhtar P., Zhu Q., Xiao Y., Han G., Santabarbara S., Shen J.-R., Lambrev P.H. (2021). Light-Adapted Charge-Separated State of Photosystem II: Structural and Functional Dynamics of the Closed Reaction Center. Plant Cell.

[38] Kitajima M., Butler W.L. (1975). Quenching of Chlorophyll Fluorescence and Primary Photochemistry in Chloroplasts by Dibromothymoquinone. Biochim. Biophys. Acta—Bioenerg..

[39] Genty B., Briantais J.-M., Baker N.R. (1989). The Relationship between the Quantum Yield of Photosynthetic Electron Transport and Quenching of Chlorophyll Fluorescence. Biochim. Biophys. Acta—Gen. Subj..

[40] Schreiber U., Bilger W., Neubauer C., Schulze E.-D., Caldwell M.M. (1995). Chlorophyll Fluorescence as a Nonintrusive Indicator for Rapid Assessment of In Vivo Photosynthesis BT—Ecophysiology of Photosynthesis.

[41] APHA (1989). Standard Methods for the Examination of Water and Wastewater.

[42] Strickland J., Parsons T.R. (1972). A Practical Handbook of Seawater Analysis.

[43] Gottardi M., Birch M.R., Dalhoff K., Cedergreen N. (2017). The Effects of Epoxiconazole and A-cypermethrin on *Daphnia magna* Growth, Reproduction, and Offspring Size. Environ. Toxicol. Chem..

[44] Ma Y., Li M., Wu M., Li Z., Liu X. (2015). Occurrences and Regional Distributions of 20 Antibiotics in Water Bodies during Groundwater Recharge. Sci. Total Environ..

[45] Bartelt-Hunt S., Snow D.D., Damon-Powell T., Miesbach D. (2011). Occurrence of Steroid Hormones and Antibiotics in Shallow Groundwater Impacted by Livestock Waste Control Facilities. J. Contam. Hydrol..

[46] Valcárcel Y., González Alonso S., Rodríguez-Gil J.L., Gil A., Catalá M. (2011). Detection of Pharmaceutically Active Compounds in the Rivers and Tap Water of the Madrid Region (Spain) and Potential Ecotoxicological Risk. Chemosphere.

[47] Agunbiade F.O., Moodley B. (2014). Pharmaceuticals as Emerging Organic Contaminants in Umgeni River Water System, KwaZulu-Natal, South Africa. Environ. Monit. Assess..

[48] Battaglin W.A., Furlong E.T., Burkhardt M.R., Peter C.J. (2000). Occurrence of Sulfonylurea, Sulfonamide, Imidazolinone, and Other Herbicides in Rivers, Reservoirs and Ground Water in the Midwestern United States, 1998. Sci. Total Environ..

[49] Loos R., Locoro G., Comero S., Contini S., Schwesig D., Werres F., Balsaa P., Gans O., Weiss S., Blaha L. (2010). Pan-European Survey on the Occurrence of Selected Polar Organic Persistent Pollutants in Ground Water. Water Res..

[50] Droz B., Drouin G., Lohmann J., Guyot B., Imfeld G., Payraudeau S. (2026). How Does Integrating Multi-Scale Monitoring and Compound-Specific Isotope Analysis Improve the Evaluation of S-Metolachlor Degradation in Agro-Ecosystems?. Hydrol. Earth Syst. Sci..

[51] Milić N., Milanović M., Letić N.G., Sekulić M.T., Radonić J., Mihajlović I., Miloradov M.V. (2013). Occurrence of Antibiotics as Emerging Contaminant Substances in Aquatic Environment. Int. J. Environ. Health Res..

[52] European Commission Commission Implementing Regulation (EU) 2024/20 of 12 December 2023 Concerning the Non-Renewal of the Approval of the Active Substance S-Metolachlor. Off. J. Eur. Union.

[53] OECD (2001). Environmental Indicators for Agriculture, Volume 3—Methods and Results.

[54] European Commission Regulation (EC) (2008). No 1272/2008 of the European Parliament and of the Council of 16 December 2008 on Classification, Labelling and Packaging of Substances and Mixtures. Off. J. Eur. Union.

[55] Guo J., Ma Z., Peng J., Mo J., Li Q., Guo J., Yang F. (2021). Transcriptomic Analysis of *Raphidocelis subcapitata* Exposed to Erythromycin: The Role of DNA Replication in Hormesis and Growth Inhibition. J. Hazard. Mater..

[56] Machado M.D., Soares E. (2020). V Reproductive Cycle Progression Arrest and Modification of Cell Morphology (Shape and Biovolume) in the Alga *Pseudokirchneriella subcapitata* Exposed to Metolachlor. Aquat. Toxicol..

[57] Lanasa S., Niedzwiecki M., Reber K.P., East A., Sivey J.D., Salice C.J. (2022). Comparative Toxicity of Herbicide Active Ingredients, Safener Additives, and Commercial Formulations to the Nontarget Alga *Raphidocelis subcapitata*. Environ. Toxicol. Chem..

[58] Machado M.D., Soares E. (2023). V Palmelloid-like Phenotype in the Alga *Raphidocelis subcapitata* Exposed to Pollutants: A Generalized Adaptive Strategy to Stress or a Specific Cellular Response?. Aquat. Toxicol..

[59] Yamagishi T., Horie Y., Tatarazako N. (2017). Synergism between Macrolide Antibiotics and the Azole Fungicide Ketoconazole in Growth Inhibition Testing of the Green Alga *Pseudokirchneriella subcapitata*. Chemosphere.

[60] Yang L.-H., Ying G.-G., Su H.-C., Stauber J.L., Adams M.S., Binet M.T. (2008). Growth-Inhibiting Effects of 12 Antibacterial Agents and Their Mixtures on the Freshwater Microalga *Pseudokirchneriella subcapitata*. Environ. Toxicol. Chem..

[61] Wu F., Pan X., Zhou Y., Zhu Y., Liu K., Li W., Han J. (2025). The Key Molecular Mechanisms of Antagonism Induced by Combined Exposure to Erythromycin and Roxithromycin in *Chlorella pyrenoidosa*. Aquat. Toxicol..

[62] Rioboo C., González O., Herrero C., Cid A. (2002). Physiological Response of Freshwater Microalga (*Chlorella vulgaris*) to Triazine and Phenylurea Herbicides. Aquat. Toxicol..

[63] Stachowski-Haberkorn S., Jérôme M., Rouxel J., Khelifi C., Rincé M., Burgeot T. (2013). Multigenerational Exposure of the Microalga *Tetraselmis suecica* to Diuron Leads to Spontaneous Long-Term Strain Adaptation. Aquat. Toxicol..

[64] Mansano A.S., Moreira R.A., Dornfeld H.C., Freitas E.C., Vieira E.M., Sarmento H., Rocha O., Seleghim M.H.R. (2017). Effects of Diuron and Carbofuran and Their Mixtures on the Microalgae *Raphidocelis subcapitata*. Ecotoxicol. Environ. Saf..

[65] Ramachandran P., Pandey N.K., Yadav R.M., Suresh P., Kumar A., Subramanyam R. (2023). Photosynthetic Efficiency and Transcriptome Analysis of *Dunaliella salina* under Hypersaline: A Retrograde Signaling Mechanism in the Chloroplast. Front. Plant Sci..

[66] Schiphorst C., Bassi R., Larkum A.W.D., Grossman A.R., Raven J.A. (2020). Chlorophyll-Xanthophyll Antenna Complexes: In between Light Harvesting and Energy Dissipation. Photosynthesis in Algae: Biochemical and Physiological Mechanisms, Advances in Photosynthesis and Respiration.

[67] Hashimoto H., Uragami C., Cogdell R.J., Stange C. (2016). Carotenoids and Photosynthesis. Carotenoids in Nature: Biosynthesis, Regulation and Function.

[68] Ricart M., Barceló D., Geiszinger A., Guasch H., de Alda M.L., Romaní A.M., Vidal G., Villagrasa M., Sabater S. (2009). Effects of Low Concentrations of the Phenylurea Herbicide Diuron on Biofilm Algae and Bacteria. Chemosphere.

[69] Girolomoni L., Bellamoli F., de la Cruz Valbuena G., Perozeni F., D’Andrea C., Cerullo G., Cazzaniga S., Ballottari M. (2020). Evolutionary Divergence of Photoprotection in the Green Algal Lineage: A Plant-like Violaxanthin de-Epoxidase Enzyme Activates the Xanthophyll Cycle in the Green Alga *Chlorella vulgaris* Modulating Photoprotection. New Phytol..

[70] Kim M., Cazzaniga S., Jang J., Pivato M., Kim G., Ballottari M., Jin E. (2024). Photoautotrophic Cultivation of a *Chlamydomonas Reinhardtii* Mutant with Zeaxanthin as the Sole Xanthophyll. Biotechnol. Biofuels Bioprod..

[71] Rousseaux C.S., Gregg W.W. (2014). Interannual Variation in Phytoplankton Primary Production at a Global Scale. Remote Sens..

[72] Falkowski P.G., Raven J.A. (2007). Aquatic Photosynthesis.

[73] Murata N., Takahashi S., Nishiyama Y., Allakhverdiev S.I. (2007). Photoinhibition of Photosystem II under Environmental Stress. Biochim. Biophys. Acta—Bioenerg..

[74] Lam M.K., Lee K.T., Mohamed A.R. (2012). Current Status and Challenges on Microalgae-Based Carbon Capture. Int. J. Greenh. Gas Control.

[75] You T., Yang Y., Cao T., Wang L., Li X. (2025). Algal Carbon Concentrating Drives Fatty Acid Biosynthesis beyond Photosynthesis. Cell Rep..

[76] Omokaro G.O., Nafula Z.S., Iloabuchi N.E., Chikukula A.A., Osayogie O.G., Nnoli E.C. (2025). Microalgae as Biofactories for Sustainable Applications: Advancing Carbon Sequestration, Bioenergy, and Environmental Remediation. Sustain. Chem. Clim. Action.

