# Supplementary material for: Beyond Single-Pollutant Toxicity: How Erythromycin–Metolachlor Mixtures Affect the Physiology of Raphidocelis subcapitata
Source: Toxics. 2026 Jul 23;14(8):645. doi: 10.3390/toxics14080645 (PMC13517234; doi:10.3390/toxics14080645)
Supplement: Supplementary file 1 [file toxics-14-00645-s001.zip › toxics-4362254-supplementary.pdf]

Toxics

SUPPLEMENTARY MATERIAL

Beyond Single-Pollutant Toxicity: How Erythromycin-Metolachlor Mixtures, Affect the physiology of *Raphidocelis subcapitata*

Beatriz C. Rocha [1], Manuela D. Machado [1, *] and Eduardo V. Soares [1,2,3, *]

[1] CIETI-Bioengineering Laboratory, ISEP, Polytechnic of Porto, rua Dr. António Bernardino de Almeida, 4249-015 Porto, Portugal
[2] CEB - Centre of Biological Engineering, University of Minho, Campus de Gualtar, 4710-057 Braga, Portugal
[3] LABBELS – Associate Laboratory, Braga/Guimarães, Portugal

***** Correspondence: mmmachado@net.sapo.pt; evs@isep.ipp.pt

Beatriz C. Rocha, email: bea.rocha2000@hotmail.com
Manuela D. Machado, email: mmmachado@net.sapo.pt; ORCID: 000-0003-3519-8654
Eduardo V. Soares; email: evs@isep.ipp.pt; ORCID: 000-0003-2280-5291

CONTENTS

Figure S1. Dose-response curves of *R. subcapitata* exposed to erythromycin (ERT) or metolachlor (MET). S2
Table S1. One-way ANOVA results (F-values and p-values) for the effects of ERY-MET mixtures on the physiological parameters of *R. subcapitata*........ S2
Table S2. Effect concentrations (EC) of ERY and MET on *R. subcapitata* S3
Table S3. Environmental concentrations of ERY and MET in natural waters........ S3
References S4

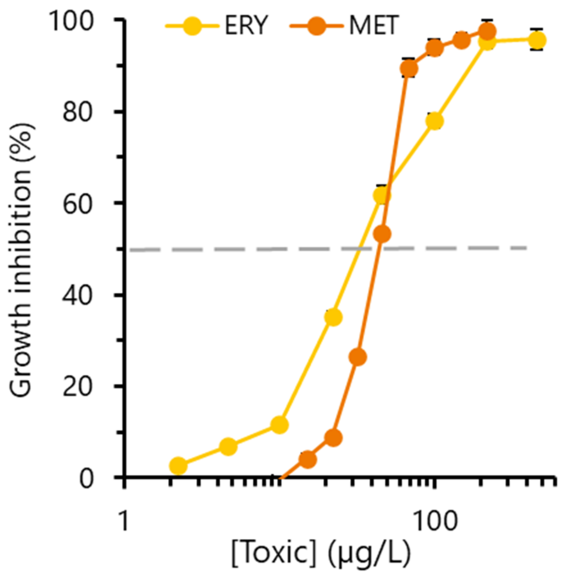

Fig S1. Dose-response curves of *R. subcapitata* exposed to erythromycin (ERY) or metolachlor (MET). Data points represent the mean ± standard deviation (error bars) of two independent experiments performed in duplicate (n=4). Where not visible, error bars are contained within the symbols.

Table S1. One-way ANOVA results (F-values and p-values) for the effects of ERY-MET mixtures on the physiological parameters of *R. subcapitata*.

Parameter	Mixture	*F*-value	*p*-value
Growth	E17+M30	24.70	<0.001
	E35+M30	42.61	<0.001
	E35+M45	51.98	<0.001
F_v/F_m	E17+M22	92.43	<0.001
	E17+M30	78.09	<0.001
	E17+M45	52.55	<0.001
	E35+M22	96.01	<0.001
Φ_{PSII}	E17+M45	32.22	<0.001
ETR	E17+M45	32.22	<0.001
Chl *a*	E17+M22	27.89	<0.001
	E17+M30	49.28	<0.001
	E35+M22	47.52	<0.001
	E35+M45	61.37	<0.001
CAR	E17+M22	14.87	<0.001
	E17+M30	31.43	<0.001
	E35+M22	46.38	<0.001
	E35+M30	21.61	<0.001
	E35+M45	25.62	<0.001

For each mixture, one-way ANOVA compared the control, the corresponding individual ERY concentration, the corresponding individual MET concentration, and the respective ERY–MET mixture. Pairwise comparisons were subsequently performed using the Tukey-Kramer multiple comparisons test.

Table S2. Effect concentrations (EC) of ERY and MET on *R. subcapitata*.

	72h-EC (μg/L)				
	10	25	50	75	90
Erythromycin	8 [4−12]	17 [15−19]	35 [32−39]	89 [83−97]	183 [176-188]
Metolachlor	22 [16−24]	31 [28−33]	44 [42−46]	59 [57−60]	70 [68−75]

EC_{10}, EC_{25}, EC_{50}, EC_{75}, and EC_{90}, correspond to the concentrations of each compound that cause 10, 25, 50, 75, and 90 % inhibition of *R. subcapitata* growth, after 72 h exposure, respectively, when compared with the control (cells not exposed to toxics). Values are presented as mean with the corresponding 95% confidence interval (in parenthesis), from two independent experiences performed in duplicate (*n*=4).

Table S3. Environmental concentrations of ERY and MET in natural waters.

**Compound**	**Source**	**Concentration range (μg/L)**	**Country**	**Reference**
**Erythromycin**				
	Groundwater	ND – 0.143	China	[44]
	Groundwater	ND – 2.38	USA	[45]
	Surface water	<0.001 – 3.85	Spain	[46]
	Surface water	up to 75.5	China	[4]
	Surface water	0.6 – 22.6	South Africa	[47]
**Metolachlor**				
	Groundwater	<0.05 – 15	USA	[9]
	Groundwater	<0.002 – 0.557	USA	[48]
	Groundwater	0.003 – 0.209	Europe	[49]
	Surface water	0.02 – 54.6	France	[50]
	Surface water	0.01 – 464	USA	[9]
	Surface water	>0.01 – 143	USA	[9]

ND: Not detected

References

4. Schafhauser, B.H.; Kristofco, L.A.; de Oliveira, C.M.R.; Brooks, B.W. Global Review and Analysis of Erythromycin in the Environment: Occurrence, Bioaccumulation and Antibiotic Resistance Hazards. *Environ. Pollut.* **2018**, *238*, 440–451, doi:10.1016/j.envpol.2018.03.052.
9. US-EPA *Draft Ecological Risk Assessment for Registration Review for Metolachlor/S-Metolachlor: EPA–HQ–OPP–2014–0772. U S Environmental Protection Agency, Washinghton DC. Https://Www.Regulations.Gov/Document?D=EPA-HQ-OPP-2014-0772-0028 [Accessed 15 December 2025]*; **2019.**
44. Ma, Y.; Li, M.; Wu, M.; Li, Z.; Liu, X. Occurrences and Regional Distributions of 20 Antibiotics in Water Bodies during Groundwater Recharge. *Sci. Total Environ.* **2015**, *518–519*, 498–506, doi:10.1016/j.scitotenv.2015.02.100.
45. Bartelt-Hunt, S.; Snow, D.D.; Damon-Powell, T.; Miesbach, D. Occurrence of Steroid Hormones and Antibiotics in Shallow Groundwater Impacted by Livestock Waste Control Facilities. *J. Contam. Hydrol.* **2011**, *123*, 94–103, doi:10.1016/j.jconhyd.2010.12.010.
46. Valcárcel, Y.; González Alonso, S.; Rodríguez-Gil, J.L.; Gil, A.; Catalá, M. Detection of Pharmaceutically Active Compounds in the Rivers and Tap Water of the Madrid Region (Spain) and Potential Ecotoxicological Risk. *Chemosphere* **2011**, *84*, 1336–1348, doi:10.1016/j.chemosphere.2011.05.014.
47. Agunbiade, F.O.; Moodley, B. Pharmaceuticals as Emerging Organic Contaminants in Umgeni River Water System, KwaZulu-Natal, South Africa. *Environ. Monit. Assess.* **2014**, *186*, 7273–7291, doi:10.1007/s10661-014-3926-z.
48. Battaglin, W.A.; Furlong, E.T.; Burkhardt, M.R.; Peter, C.J. Occurrence of Sulfonylurea, Sulfonamide, Imidazolinone, and Other Herbicides in Rivers, Reservoirs and Ground Water in the Midwestern United States, 1998. *Sci. Total Environ.* **2000**, *248*, 123–133, doi:10.1016/S0048-9697(99)00536-7.
49. Loos, R.; Locoro, G.; Comero, S.; Contini, S.; Schwesig, D.; Werres, F.; Balsaa, P.; Gans, O.; Weiss, S.; Blaha, L.; et al. Pan-European Survey on the Occurrence of Selected Polar Organic Persistent Pollutants in Ground Water. *Water Res.* **2010**, *44*, 4115–4126, doi:10.1016/j.watres.2010.05.032.
50. Droz, B.; Drouin, G.; Lohmann, J.; Guyot, B.; Imfeld, G.; Payraudeau, S. How Does Integrating Multi-Scale Monitoring and Compound-Specific Isotope Analysis Improve the Evaluation of S-Metolachlor Degradation in Agro-Ecosystems? *Hydrol. Earth Syst. Sci.* **2026**, *30*, 1291–1307, doi:10.5194/hess-30-1291-2026.
